# Study on the Mechanism of Ginseng in the Treatment of Lung Adenocarcinoma Based on Network Pharmacology

**DOI:** 10.1155/2020/2658795

**Published:** 2020-07-31

**Authors:** Qiu-Yue Li, Cheng-Zhi Hou, Li-Ping Yang, Xue-Lei Chu, Yuan Wang, Ping Zhang, Yong Zhao

**Affiliations:** ^1^Wang Jing Hospital, China Academy of Chinese Medical Sciences, Beijing 100102, China; ^2^Institute of Chinese Materia Medica, China Academy of Chinese Medical Sciences, Beijing 100700, China

## Abstract

**Background:**

Ginseng, a traditional Chinese medicine, was used to prevent and treat many diseases such as diabetes, inflammation, and cancer. In recent years, there are some reports about the treatment of lung adenocarcinoma with ginseng monomer compounds, but there is no systematic study on the related core targets and mechanism of ginseng in the treatment of lung adenocarcinoma up to now. Therefore, this study systematically and comprehensively studied the molecular mechanism of ginseng in the treatment of lung adenocarcinoma based on network pharmacology and further proved the potential targets by A549 cell experiments for the first time.

**Methods:**

The targets of disease and drug were obtained from Gene database. Subsequently, the compound-target network was constructed, and the core potential targets were screened out by plug-in into Cytoscape. Furthermore, the core targets and mechanism of ginseng in the treatment of lung adenocarcinoma were verified by MTT test, cell scratch test, immunohistochemistry, and qRT-PCR.

**Results:**

1791 disease targets and 144 drug targets were obtained by searching the Gene database. Meanwhile, 15 core targets were screened out: JUN, MAPK8, PTGS2, CASP3, VEGFA, MMP9, AKT1, TNF, FN1, FOS, MMP782, IL-1*β*, IL-2, ICAM1, and HMOX1. The results of cell experiments indicate that ginseng could treat lung adenocarcinoma by cell proliferation, migration, and apoptosis. In addition, according to the results of the 15 core targets by qRT-PCR, JUN, IL-1*β*, IL-2, ICAM1, HMOX1, MMP9, and MMP2 are upregulated core targets, while PTGS2 and TNF are downregulated core targets.

**Conclusion:**

This study systematically and comprehensively studied 15 core targets by network pharmacology for the first time. Subsequently, it is verified that 9 core targets for ginseng treatment of lung adenocarcinoma, namely, JUN, IL-1*β*, IL-2, ICAM1, HMOX1, MMP9, MMP2, PTGS2, and TNF, are closely related to the proliferation, migration, and apoptosis of lung adenocarcinoma cells. This study has reference value for the clinical application of ginseng in the treatment of lung adenocarcinoma.

## 1. Introduction

Cancer is the major cause of death worldwide, among which lung cancer is the top killer [1, 2]. Lung adenocarcinoma, which belongs to non-small cell lung cancer, is the most common type of lung cancer, accounting for 40% of all lung cancer cases [3]. According to the stage of lung cancer, there are treatments including surgery, radiation therapy, chemotherapy, targeted therapy, and immunotherapy [4]. However, there is no significant improvement in cancer mortality and 5-year relative survival for all cancers in the past 20 years [5]. Therefore, it is critical to discover a new strategy for cancer treatment.

Traditional Chinese medicine, as one of the popular alternative treatments for lung cancer, can enhance physical function, reduce the adverse effects of chemotherapy, and improve long-term survival [6]. Ginseng, the root of *Panax ginseng* C. A. Mey. (Araliaceae), is a precious and common Chinese traditional herb. It was first recorded by Shennong Bencao Jing and has thousands of years of history [7]. Ginseng is generally known as tonic drug for promoting longevity and widely used in China, South Korea, Japan, and other Far East countries [8]. According to pharmacological research, the bioactivities of ginseng, including antiaging activity, antidiabetic activity, immunoregulatory activity, anticancer activity, neuroregulation activity, and wound and ulcer healing activity, mainly stem from ginsenoside, polysaccharide, alkaloids, glucosides, phenolic acid, and other ingredients [9, 10]. In recent years, there are some reports about the treatment of lung adenocarcinoma with ginseng monomer compounds such as ginsenoside and polysaccharide [11, 12], but there is no systematic and comprehensive study on the related core targets and mechanism of ginseng in the treatment of lung adenocarcinoma.

Network pharmacology, with the characteristics of “network target, multicomponent” mode, provides an effective way to evaluate polypharmacological effects and anticancer molecular mechanisms of drugs [13]. At present, the mechanisms of multicomponent, multitarget, and multipath treatment of lung adenocarcinoma by ginseng are unclear. Therefore, this paper systematically and comprehensively reveals the molecular mechanism of ginseng in the treatment of lung adenocarcinoma based on network pharmacology and carries out A549 cell experiments for preliminary confirmation. Our study confirms that ginseng (with main components including total ginsenoside and ginseng polysaccharide) is a rational and alternative strategy to treat lung adenocarcinoma, and upregulated JUN, IL-1*β*, IL-2, ICAM1, HMOX1, MMP9, and MMP2 targets and downregulated PTGS2 and TNF genes may be predictably important targets related to cell proliferation/apoptosis/migration and poor prognosis of cancer for ginseng treatment of lung adenocarcinoma.

## 2. Materials and Methods

### 2.1. Screening Active Compounds

All the compounds of ginseng were obtained from the TCMSP database (Traditional Chinese Medicine Systems Pharmacology, http://www.ibts.hkbu.eduhk/LSP/tcmsp.php) [14]. The potential active compounds of ginseng were screened according to Oral Bioavailability (OB) ≥ 0.3 and Drug-Like (DL) Index ≥0.18. In the screening process, we added additional filter criteria, Caco-2 ≥ −0.4 and half-life ≥ 8, to draw a clear direction and focus [15]. By searching the active compounds of ginseng in Chinese Pharmacopoeia (2015) and Chinese Medicine Dictionary, the important active compounds which were preliminarily excluded by TCMSP were added as candidate active compounds.

### 2.2. Access to Disease Genes and Drug Targets

The known genes related to lung adenocarcinoma were acquired from the PubMed database (update time: May 21, 2019, https://www.ncbi.nlm.nih.gov/gene). The keywords “lung adenocarcinoma” were used when searching the PubMed database, and only those genes from “*Homo sapiens*” were used as the research targets, analyzed, and discussed in this paper. In the end, 1791 disease genes were obtained.

Meanwhile, the targets of selected active compounds were obtained from TCMSP and Hit databases (Herbal Ingredients' Targets Database, URL: http://www.lifecenter.sgst.cn/hit/) [16]. After combining the two databases and excluding duplicates and nonhuman target proteins, the gene names of the targets were obtained from the UniProt database.

### 2.3. Construction of Protein-Protein Interaction Network and Screening of Key Targets

Protein-protein interaction network of acquired drug targets were constructed by the STRING database (https://string-db.org/). 28 key targets were selected according to the comprehensive analysis of the topological parameters “closeness,” “betweenness,” and “degree” [17]. Subsequently, 15 core genes were further screened out by the cytoHubba plug-in of Cytoscape [18].

### 2.4. Construction and Analysis of Biological Network

Since ginseng involves a large number of targets and active compounds, the “compound-target” network of 28 key targets was constructed to explore the relationship between active compounds and targets. Meanwhile, to further clarify the biological function of the 28 key targets in ginseng, a GO enrichment analysis for “compound-target” network was carried out with Cytoscape. In addition, enrichment analyses of KEGG pathway were carried out with KOBAS (http://kobas.cbi.pku.edu.cn/)database. GO enrichment analysis of key targets and enrichment analysis of KEGG pathways further explained the potential mechanism for the treatment of lung adenocarcinoma with ginseng.

### 2.5. Experiment Verification

#### 2.5.1. Cell Culture

A549 cells were purchased from ATCC and grown in DMEM medium (HyClone, USA) supplemented with 10% fetal bovine serum (FBS) (Gibco, USA) and 100 U/ml penicillin-streptomycin. All cells were cultured as monolayers and maintained in a cell culture incubator at 37°C and 5% CO_2_. Previous literature research revealed that the main active components of ginseng in the treatment of lung adenocarcinoma were ginsenosides (such as ginsenoside Rh2/Rh3) and polysaccharides [19–21], with ginsenoside and polysaccharide used as the medicated group and cyclophosphamide used as the positive control group in the cell experiments.

#### 2.5.2. Cell Proliferation Assay

The cells proliferation was detected by 3-(4,5-dimethylthiazol-2-yl)-2,5-diphenyltetrazolium bromide (MTT) test, taking ginsenoside, polysaccharide, and cyclophosphamide as the different experimental groups and A549 cells as the blank control group. The cell density was adjusted to 5 × 103 cells/ml. Next, the cells were transferred to 96-well plate and cultured for 24 hours. Subsequently, the culture medium was removed, and 100 *μ*L of the serum-free culture medium containing different concentrations of ginsenoside, polysaccharide, and cyclophosphamide was added. MTT solution (5 mg/mL, Solarbio, China) was placed into every well of the 96-well plate after 24 hours and cultured for 4 hours. The culture medium was removed and 100 *μ*l DMSO (per well) was added. An ELISA meter was used to examine the absorbance of each pore at the wavelength of 630 nm.

#### 2.5.3. Cell Migration Assay

Cell migration assay was performed using the scratch test. In this trial, ginsenoside and polysaccharide were used as the different experimental groups, and A549 cells as the blank control group. A549 cells were seeded on a six‐well plate. When the cells were cultured to 80% confluence, scratches were made using a 10 *μ*l pipette tip. Subsequently, the culture medium was discarded and the cells were washed with PBS. Next, the cells were added to the serum-free culture medium containing the best concentrations of ginsenoside and polysaccharide. Next, the cells were photographed under the microscope after 0, 6, and 24 hours.

#### 2.5.4. Immunocytochemistry

The effect of ginsenoside and polysaccharide on A549 cells apoptosis was detected by immunocytochemistry. In this trial, ginsenoside and polysaccharide were used as the different experimental groups, and A549 cells as the blank control group. The A549 cells were seeded into 24-well plate with a concentration of 1 × 10^5^ and cultured for 24 hours. Subsequently, the serum-free culture medium containing best concentrations of ginsenoside and polysaccharide was added, and then culture continued for 24 hours. The cells were fixed with paraformaldehyde for 20 min at 4°C and washed with PBS. Next, after being incubated with H_2_O_2_ for 10 min at room temperature, the cells were added to anti-rabbit Caspase-9 primary antibody (BOSTER, China) for 60 min at 37°C. Subsequently, the cells were incubated with anti-rabbit secondary antibody (ZsBio, China), after being washed with PBS, and then observed with DAB (ZsBio, China).

#### 2.5.5. qRT-PCR for mRNA Expression Levels

The mRNA expression levels of 15 core targets were detected by qRT-PCR. In this trial, ginsenoside and polysaccharide were used as the different experimental groups, and A549 cells as the blank control group. After being placed into a six‐well plate, the A549 cells were add to culture medium containing best concentrations of ginsenoside and polysaccharide and then cultured for 24 hours. Next, the total RNA, was extracted using the Trizol reagent (TIANGEN, China), whose concentration and purity were detected using NanoDrop One nucleic acid quantizer. According to the manufacturer's protocol, the RNA was reverse-transcribed into cDNA using the extracted RNA sample as a template. As shown in Table 1, the primer sequences were designed in the laboratory and synthesized by Sangon Biotech (Sangon, China) based on the mRNA sequences obtained from the NCBI database. Subsequently, qPCR was performed on an ABI 7500 system (Applied Bioscience). mRNA expression was presented as the fold change relative *β*-actin and was determined using the 2^−∆∆*Ct*^ method.

#### 2.5.6. Statistical Analysis

All quantitative data were presented as mean ± standard deviation from at least three independent experiments. Statistical analysis was performed using SPSS version 25.0 (IBM Corp.). Differences between the two groups were determined using Student's *t*-test analysis. Comparisons between ≥3 groups were performed using a one-way ANOVA with a post hoc Dunnett's test. ^*∗*^*p* < 0.05 and ^*∗∗*^*p* < 0.01 were considered to have statistical significance between the treatment group and the control group.

## 3. Results

### 3.1. Disease Genes, Drug Targets, and Effective Active Compounds

1791 disease targets and 144 drug targets (including 15 core targets, namely, JUN, MAPK8, PTGS2, CASP3, VEGFA, MMP9, AKT1, TNF, FN1, FOS, MMP2, IL-1*β*, IL-2, ICAM1, and HMOX1) were obtained by searching Gene database. According to active compounds corresponding to the targets, ginsenoside Rh2, ginsenoside Rh4_qt, ginsenoside Rf, ginsenoside Rd, ginsenoside Re, panaxydol, stigmasterol, beta-sitosterol, and kaempferol were screened out from ginseng.

### 3.2. Construction of Compound-Target Network

**Figure 1:**
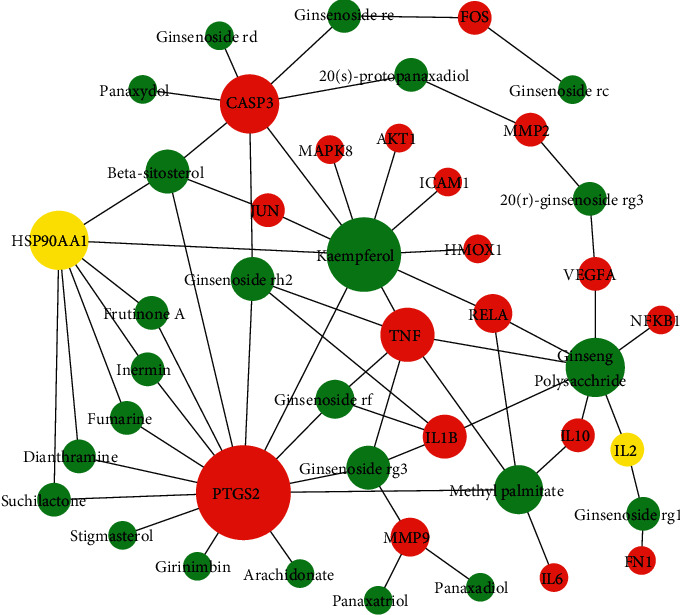
Network relationship of key targets and active compounds of ginseng. Green represents the active compounds of ginseng, red represents lung adenocarcinoma genes, and yellow represents non-lung adenocarcinoma genes.

As shown in Figure 1, the result showed the network relationship of the 28 key targets and the active compounds of ginseng. In this network, the larger the node is, the more compounds the target has. It was found that there are 14 active compounds targeted at PTGS2 in this study. In addition, there are 7 active compounds targeted at CASP3, 6 at TNF, 4 at IL-1*β*, and 3 at MMP9. The rest are lung adenocarcinoma genes except IL-2 and HSP90AA1 in the 28 key targets. According to Figure 1, JUN, MAPK8, PTGS2, CASP3, VEGFA, MMP9, ICAM1, HMOX1, and so on were the key targets for ginseng treatment of lung adenocarcinoma. Among the targets, VEGFA can promote cell migration and inhibit cell apoptosis. MAPK/ILs/TNF could influence inflammatory response. Interleukin-1 beta (IL-1*β*) drives tumor growth, invasion, and metastasis. MMPs play an important role in tumorigenesis, cancer cell migration, cell growth, and angiogenesis. The functions of these targets are explained in detail in the Discussion section. According to the functions of these targets, ginseng could treat lung adenocarcinoma through cell differentiation, proliferation, apoptosis, migration, and inflammatory response. This is also consistent with the multitarget and multipathway mechanism of traditional Chinese medicine.

### 3.3. GO Enrichment Analysis

**Figure 2:**
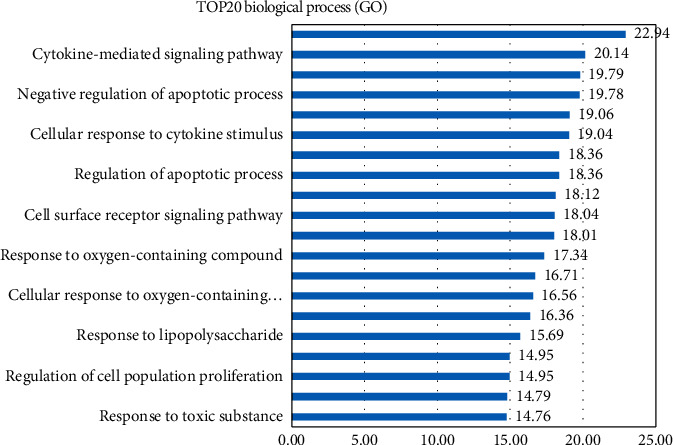
The functional analysis for identified compounds-related targets. The docking targets-related GO terms.

The results of the GO enrichment analysis consist of biological processes (BP), cell components (CC), and molecular functions (MF). According to FDR, the top 20 biological processes of the 28 key targets are shown in Figure 2, including cellular response to organic substance, cytokine-mediated signaling pathway, response to cytokine, and negative regulation of apoptotic process.

### 3.4. KEGG Pathways Enrichment Analysis

**Figure 3:**
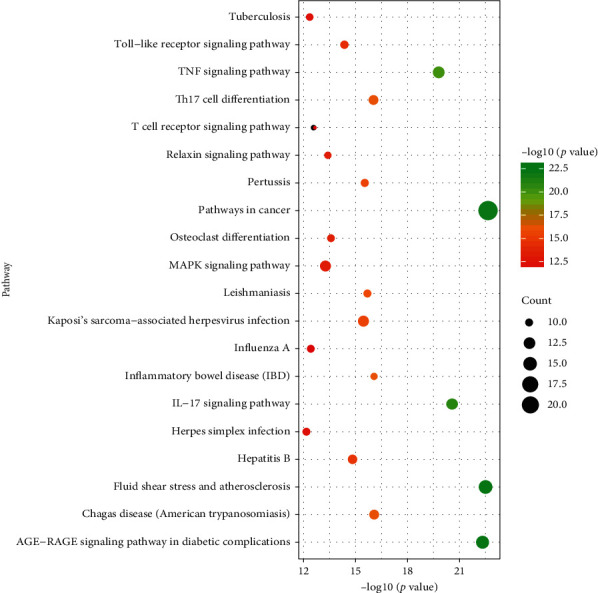
Functional analysis for identified compounds-related targets. The docking targets-related KEGG pathways distribution.

To further study the biological function of key targets, enrichment analysis of KEGG pathway was carried out for key targets. Meanwhile, the top 20 signaling pathways of key targets, including pathways in cancer, fluid shear stress and atherosclerosis, AGE-RAGE signaling pathway in diabetic complications, IL-17 signaling pathway, and TNF signaling pathway, are shown in Figure 3.

### 3.5. Ginsenoside and Polysaccharide Can Inhibit Proliferation and Promote Apoptosis of Lung Adenocarcinoma Cells

**Figure 4:**
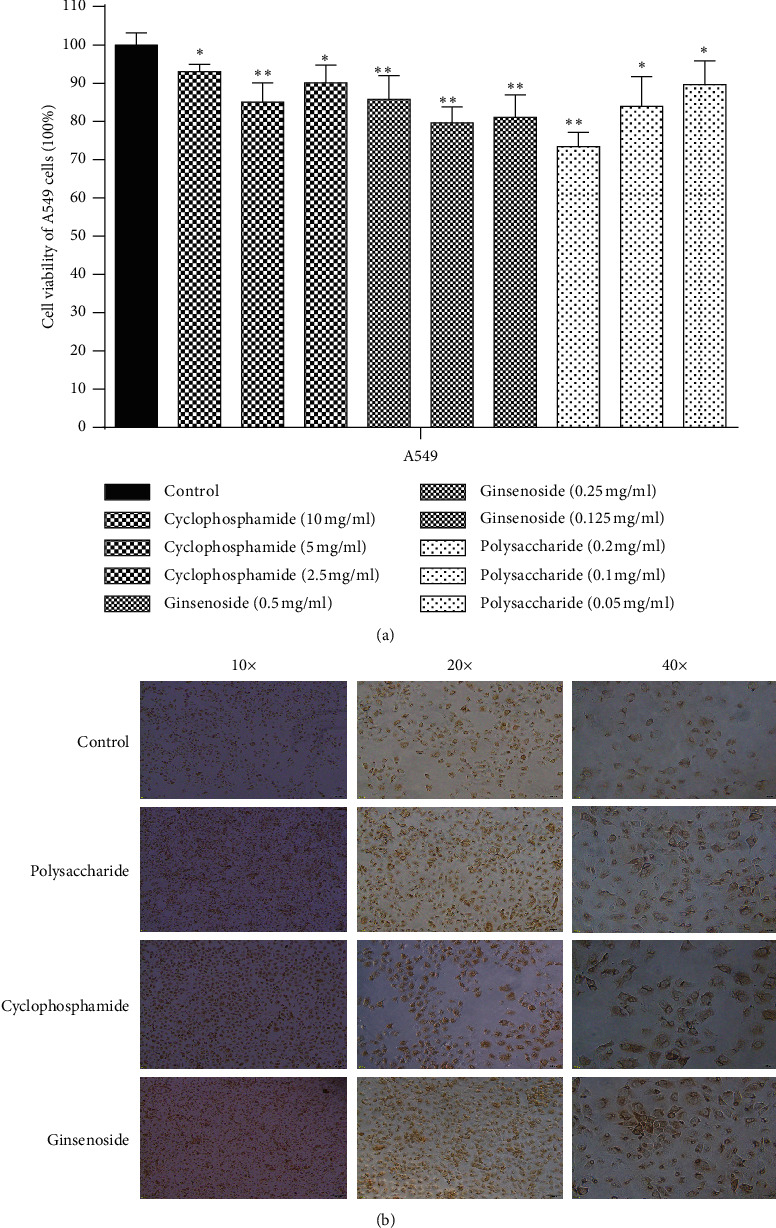
The effect of ginseng on the proliferation and apoptosis of lung adenocarcinoma. (a) MTT test result (control group, cyclophosphamide group, ginsenoside group, polysaccharide group) (^*∗*^*p* < 0.05, ^*∗∗*^*p* < 0.01). (b) Immunocytochemistry result of apoptosis factor (10×, 20×, 40×).

Cell proliferation was detected by MTT test. As shown in Figure 4(a), the result showed that different concentrations of ginsenoside, polysaccharide, and cyclophosphamide could inhibit the proliferation of A549 cells (*p* < 0.05). However, with the increase of drug concentration, the cell viability of polysaccharide group decreased. Therefore, 0.2 mg/ml was selected as the best concentration of polysaccharide in our following experiments. Meanwhile, 0.25 mg/ml ginsenoside and 5 mg/ml cyclophosphamide had obvious influences on proliferation of A549 cells and were considered the best concentrations. In addition, considering that the decrease in cell proliferation was likely to be associated with apoptosis, the expression of apoptosis factor (Caspase-9) was detected by immunocytochemistry. As shown in Figure 4(b), the expression of Caspase-9 in ginsenoside and polysaccharide group is more obvious compared with the control group. In conclusion, the above results reveal that ginsenoside and polysaccharide inhibit the proliferation of lung adenocarcinoma cells and promote apoptosis by affecting the expression of Caspase-9.

### 3.6. Ginsenoside Can Inhibit the Migration of Lung Adenocarcinoma Cells

**Figure 5:**
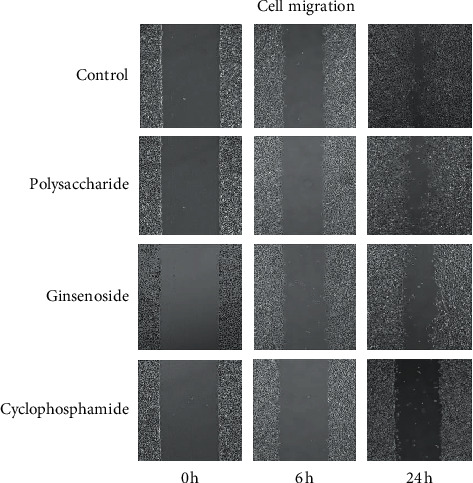
The effect of ginseng on the migration of lung adenocarcinoma cells (0 h, 6 h, 24 h).

The effect of ginsenoside and polysaccharide on cell migration was assessed using the scratch test. As shown in Figure 5, the result showed that migration rate of A549 cells decreased in the ginsenoside group compared with the control group over time. However, the migration rate of A549 cells had no obvious change in the polysaccharide group. In conclusion, these results indicate that ginsenosides could inhibit migration of lung adenocarcinoma cells.

### 3.7. Validating the Core Targets of Ginsenoside and Polysaccharide

**Figure 6:**
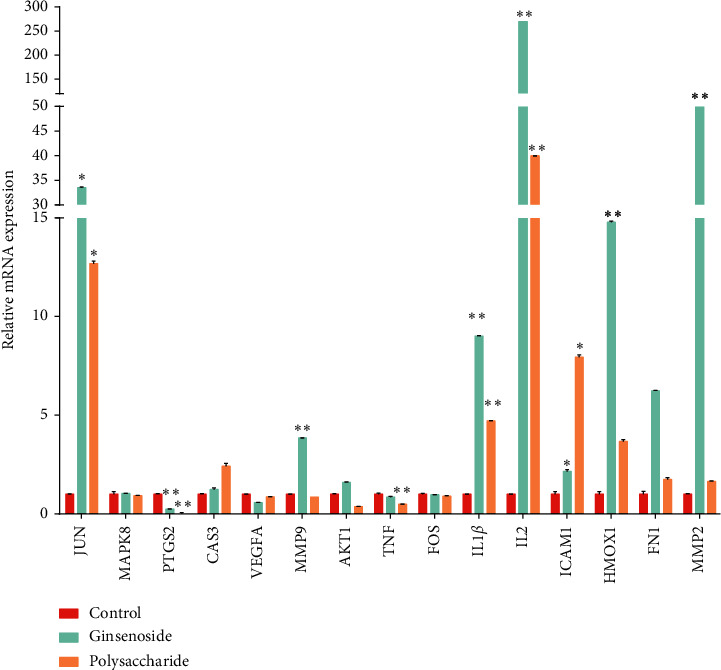
The mRNA expression levels of 15 core targets. Compared with the control group, the expression levels of JUN, IL-1*β*, IL-2, ICAM1, HMOX1, MMP9, MMP2, PTGS2, and TNF mRNA in the administration group have significant difference in the treatment group (^*∗*^*p* < 0.05, ^*∗∗*^*p* < 0.01).

To determine whether the core targets play important roles in ginsenoside and polysaccharide treatment of lung adenocarcinoma, the mRNA expression levels of 15 core targets were detected by qRT-PCR from the blank control group, ginsenoside group, and polysaccharide group. As shown in Figure 6, the mRNA expression levels of JUN, IL-1*β*, IL-2, HMOX1, PTGS2, ICAM1, MMP9, and MMP2 were significantly different between the blank control group and ginsenoside group (*p* < 0.05). Meanwhile, JUN, PTGS2, IL-1*β*, IL-2, TNF, and ICAM1 had significant difference in polysaccharide group compared with the control group (*p* < 0.05). Furthermore, JUN, IL-1*β*, IL-2, ICAM1, HMOX1, MMP9, and MMP2 were overexpressed, while PTGS2 and TNF were underexpressed. In conclusion, the results suggested that the above core targets could play important roles in ginsenoside and polysaccharide treatment of lung adenocarcinoma.

## 4. Discussion

Ginseng, as an ancient precious herbal medicine containing a variety of effective ingredients, is widely used in the treatment of various diseases, having antitumor effect [22]. A comprehensive description of the therapeutic effect of ginseng in traditional Chinese Medicine is available in the Shen Nong's Herbal Classic, which still has guiding significance for modern research. According to the literature, ginseng can nourish the five internal organs, tranquilize the nerves, stabilize the spirit, stop palpitation with fear, exorcise pathogenic factors, brighten eyes, delight, improve intelligence, and take a long time to be healthy and macrobiotic. Furthermore, ginseng enters the lung, spleen, and heart meridians, which are related to the treatment of lung adenocarcinoma. Studies have indicated that the components of ginseng play an important role against lung adenocarcinoma [19, 23].

In this study, the targets of disease and drug were obtained from Gene database. 28 key targets were selected by topological analysis. According to compound-target network of the 28 key targets (Figure 1), ginseng, including multiple active compounds such as ginsenoside and polysaccharide, might treat lung adenocarcinoma through cell differentiation, proliferation, apoptosis, migration, and inflammatory response. In the experiments, the results of MTT test, scratch test, and immunocytochemistry confirmed that ginseng could treat lung adenocarcinoma through proliferation, apoptosis, and migration (Figures 4 and 5). To explore these 28 key targets holistically, enrichment analysis of key target genes was conducted. The results of GO and KEGG pathways enrichment analysis showed that these targets are mainly associated with signal-related pathways, such as pathways in cancer, fluid shear stress and atherosclerosis, AGE-RAGE signaling pathway in diabetic complications, IL-17 signaling pathway, and TNF signaling pathway (Figures 2 and 3). Moreover, 15 core genes, namely, JUN, MAPK8, PTGS2, CASP3, VEGFA, MMP9, AKT1, TNF, FN1, FOS, MMP2, IL-1b, IL-2, ICAM1, and HMOX1, were screened out through cytoHubba plug-in of Cytoscape based on 28 key genes. These core targets might play important roles in ginseng treatment of lung adenocarcinoma. To determine the role of 15 core targets in the treatment of lung adenocarcinoma by ginseng, qRT-PCR was performed to detect the mRNA expression levels of the 15 core targets from A549 cells in the control group, ginsenoside group, and polysaccharide. The results showed the mRNA expression levels of JUN, IL-1*β*, IL-2, ICAM1, HMOX1, MMP9, MMP2, PTGS2, and TNF had significant difference in control group, ginsenoside group, and polysaccharide group, which suggested that ginsenoside and polysaccharide might have therapeutic potential for the treatment of lung adenocarcinoma by targeting the above-mentioned core genes (Figure 6).

Among these core targets, c-Jun is a major part of AP-1 transcription factors consisting of homodimers and heterodimers of the JUN, FOS, and ATF gene family members, and it is often overexpressed in NSCLC. Kikuchi et al. [24] found that AP-1 and PI3K/Akt pathways play an important role in the growth of some NSCLC cells through experiments. Interleukin-1 beta (IL-1*β*), a potent driver of tumor progression, is highly expressed in metastatic NSCLC tumors and drives tumor growth, invasion, and metastasis [25, 26]. Petrella et al. [27] explored the effects of IL-1*β* on NSCLC cell line A549 and found IL-1*β* to be potential therapeutic target for NSCLC tumors. Interleukin-2 (IL-2), a cytokine signaling molecule necessary for the differentiation, growth, and proliferation of T-lymphocytes, has been shown to improve the survival rate of patients with NSCLC [28, 29]. It has been proved that the imbalance of the IL-2/IL-2 receptor system in advanced NSCLC represents a marker of disease with potential prognostic value [30]. In addition, it has been proved that IL-2 activation plays an essential role in the restoration of the immunocompetence of lymphocytes against lung cancer [31]. Intercellular adhesion molecule-1 (ICAM-1), a member of the immunoglobulin gene superfamily, is a single chain surface membrane glycoprotein expressed in a variety of cells [32]. It has been proved that soluble ICAM-1 (sICAM-1) facilitates the growth of tumor cell and allows the tumor cell to bypass immune recognition through combining circulating lymphocytes [33]. Several studies found that increased sICAM-1 level is related to high tumor burden and advanced disease in NSCLC [34]. Heme oxygenase-1 (HMXO-1), an antioxidant protein, has been shown to protect cells against oxidative stress. Ma [35] explored the role of HMXO-1 activation in NTP-induced apoptosis in A549 cells and found HMXO-1 to be a potential target for NTP cancer treatment. In addition, Cao [36] found that knockdown of HMOX1 inhibits the proliferation, invasion, and migration of A549 cells by impeding autophagy. Some studies have indicated that TNF-*α* plays an essential role in connecting the molecules associated with inflammation and cancer. Furthermore, clinical studies have shown that the expression level of TNF-*α* in serum samples acquired from patients with NSCLC increased with the stage of cancer [37, 38]. MMPs play an important role in tumorigenesis, cancer cell migration, cell growth, and angiogenesis [39]. MMP2 has a role in regulating the migration and invasion of NSCLC [40]. COX-2, encoded by the PTGS2 gene, a type of induction enzyme, is only produced by stimulation from associated cytokines, tumor genes, and tumor inducers [41]. It has been proved that COX-2 inhibitors have good antitumor effects [42].

## 5. Conclusions

Traditional Chinese medicine is one of the most important types of complementary and alternative medicine and contributes greatly to cancer-related disease therapy. This study proposed and applied a network pharmacology-based analysis to suggest that ginseng can be used in the treatment of lung adenocarcinoma by regulating candidate targets. Through experimental studies, we further revealed the therapeutic effects of ginseng on lung adenocarcinoma, such as suppressing A549 cell proliferation and migration and promoting apoptosis. Meanwhile, according to the results of qRT-PCR, it is speculated that ginseng can treat lung adenocarcinoma by up-regulated JUN, IL-1*β*, IL-2, ICAM1, HMOX1, MMP9, and MMP2 targets and down-regulated PTGS2 and TNF genes. Further research is in progress.

The results of this study are helpful for us to understand the molecular mechanism and have reference value for the clinical application of ginseng in the treatment of lung adenocarcinoma. Considering the clinical status of lung adenocarcinoma treatment, new therapeutic agents are urgently needed, and ginseng deserves further assessment.

## Figures and Tables

**Table 1:** The mRNA sequences of the 15 core targets obtained from the NCBI database.

Gene name	Forward primer (5′-3′)	Reverse primer (5′-3′)
MAPK8	TGAAGCAGAAGCTCCACCAC	CAGCCCTCTCCTTTAGGTGC
JUN	CAAACCTCAGCAACTTCAACC	CTGGGACTCCATGTCGATG
PTGS2	TGTCAAAACCGAGGTGTATGTA	AACGTTCCAAAATCCCTTGAAG
CASP3	CCAAAGATCATACATGGAAGCG	CTGAATGTTTCCCTGAGGTTTG
VEGFA	ATCGAGTACATCTTCAAGCCAT	GTGAGGTTTGATCCGCATAATC
MMP9	CAGTACCGAGAGAAAGCCTATT	CAGGATGTCATAGGTCACGTAG
AKT1	TGACCATGAACGAGTTTGAGTA	GAGGATCTTCATGGCGTAGTAG
TNF	CGTGGAGCTGGCCGAGGAG	GCAGGCAGAAGAGCGTGGTG
FOS	CTTCCCAGAAGAGATGTCTGTG	TGGGAACAGGAAGTCATCAAAG
IL-1*β*	GCCAGTGAAATGATGGCTTATT	AGGAGCACTTCATCTGTTTAGG
IL-2	CACCAGGATGCTCACATTTAAG	CTCCAGAGGTTTGAGTTCTTCT
ICAM1	TGCAAGAAGATAGCCAACCAAT	GTACACGGTGAGGAAGGTTTTA
HMOX1	CCTCCCTGTACCACATCTATGT	GCTCTTCTGGGAAGTAGACAG
FN1	AATAGATGCAACGATCAGGACA	GCAGGTTTCCTCGATTATCCTT
MMP2	ATTGTATTTGATGGCATCGCTC	ATTCATTCCCTGCAAAGAACAC

## Data Availability

The chemical ingredients of ginseng were extracted from TCMSP platform to support the findings of this study. The important nodes of ginseng compound-target network used to support the findings of this study are included within the article. The PPI network used to rank the importance of targets was constructed using STRING software. The gene ontology (GO) functional enrichment analysis included the top 20 cell components (CC), molecular functions (MF), biological processes (BP), and KEGG pathways used to elaborate the pharmacological mechanism of ginseng, which are included within the article. All data including experimental results used during the study appear in the submitted article.

## References

[1] McGuire S. (2016). World cancer report 2014. *Advances in Nutrition*.

[2] Torre L. A., Bray F., Siegel R. L., Ferlay J., Lortet-Tieulent J., Jemal A. (2015). Global cancer statistics, 2012. *CA: A Cancer Journal for Clinicians*.

[3] Zappa C., Mousa S. A. (2016). Non-small cell lung cancer: current treatment and future advances. *Translational Lung Cancer Research*.

[4] Lemjabbar-Alaoui H., Hassan O. U., Yang Y.-W., Buchanan P. (2015). Lung cancer: biology and treatment options. *Biochimica et Biophysica Acta (BBA)-Reviews on Cancer*.

[5] Allemani C., Weir H. K., Carreira H. (2015). Global surveillance of cancer survival 1995-2009: analysis of individual data for 25 676 887 patients from 279 population-based registries in 67 countries (CONCORD-2). *The Lancet*.

[6] Liao Y.-H., Li C.-I., Lin C.-C., Lin J.-G., Chiang J.-H., Li T.-C. (2017). Traditional Chinese medicine as adjunctive therapy improves the long-term survival of lung cancer patients. *Journal of Cancer Research and Clinical Oncology*.

[7] Hu S.-Y. (1977). A contribution to our knowledge of ginseng. *The American Journal of Chinese Medicine*.

[8] Yun T. K., Lee Y. S., Lee Y. H., Kim S. I., Yun H. Y. (2001). Anticarcinogenic effect of Panax ginseng C.A. Meyer and identification of active compounds. *Journal of Korean Medical Science*.

[9] Liu C. X., Xiao P. G. (1992). Recent advances on ginseng research in China. *Journal of Ethnopharmacology*.

[10] Ru W., Wang D., Xu Y. (2015). Chemical constituents and bioactivities of Panax ginseng (C. A. Mey.). *Drug Discoveries & Therapeutics*.

[11] Cong Z., Zhao Q., Yang B. (2020). Ginsenoside Rh3 inhibits proliferation and induces apoptosis of colorectal cancer cells. *Pharmacology*.

[12] Jeong D., Ham J., Park S. (2019). Ginsenoside Rh2 suppresses breast cancer cell proliferation by epigenetically regulating the long noncoding RNA C3orf67-AS1. *The American Journal of Chinese Medicine*.

[13] Li S., Zhang B. (2013). Traditional Chinese medicine network pharmacology: theory, methodology and application. *Chinese Journal of Natural Medicines*.

[14] Ru J., Li P., Wang J. (2014). TCMSP: a database of systems pharmacology for drug discovery from herbal medicines. *Journal of Cheminformatics*.

[15] Li Y., Zhang J., Zhang L. (2015). Systems pharmacology to decipher the combinational anti-migraine effects of Tianshu formula. *Journal of Ethnopharmacology*.

[16] Kang H., Tang K., Liu Q. (2013). HIM-herbal ingredients in-vivo metabolism database. *Journal of Cheminformatics*.

[17] Zhang Y.-Q., Guo Q.-Y., Li Q.-Y. (2018). Main active constituent identification in Guanxinjing capsule, a traditional Chinese medicine, for the treatment of coronary heart disease complicated with depression. *Acta Pharmacologica Sinica*.

[18] Chin C.-H., Chen S.-H., Wu H.-H., Ho C.-W., Ko M.-T., Lin C.-Y. (2014). cytoHubba: identifying hub objects and sub-networks from complex interactome. *BMC Systems Biology*.

[19] Yu J. S., Roh H.-S., Baek K.-H. (2018). Bioactivity-guided Isolation of Ginsenosides from Korean Red ginseng with cytotoxic activity against human lung adenocarcinoma cells. *Journal of Ginseng Research*.

[20] Chen Y., Zhang Y., Song W., Zhang Y., Dong X., Tan M. (2019). Ginsenoside Rh2 inhibits migration of lung cancer cells under hypoxia *via* mir-491. *Anti-Cancer Agents in Medicinal Chemistry*.

[21] Lin L., Cheng K., He Z. (2019). A polysaccharide from hedyotis diffusa interrupts metastatic potential of lung adenocarcinoma A549 cells by inhibiting EMT via EGFR/Akt/ERK signaling pathways. *International Journal of Biological Macromolecules*.

[22] Yun T.-K. (2001). Panax ginseng-a non-organ-specific cancer preventive?. *The Lancet Oncology*.

[23] Kim Y.-J., Choi W.-I., Jeon B.-N. (2014). Stereospecific effects of ginsenoside 20-Rg3 inhibits TGF-*β*1-induced epithelial-mesenchymal transition and suppresses lung cancer migration, invasion and anoikis resistance. *Toxicology*.

[24] Kikuchi J., Kinoshita I., Shimizu Y. (2008). Simultaneous blockade of AP-1 and phosphatidylinositol 3-kinase pathway in non-small cell lung cancer cells. *British Journal of Cancer*.

[25] Elaraj D. M., Weinreich D. M., Varghese S. (2006). The role of interleukin 1 in growth and metastasis of human cancer xenografts. *Clinical Cancer Research*.

[26] Krelin Y., Voronov E., Dotan S. (2007). Interleukin-1*β*-driven inflammation promotes the development and invasiveness of chemical carcinogen-induced tumors. *Cancer Research*.

[27] Petrella B. L., Armstrong D. A., Vincenti M. P. (2012). Interleukin-1 beta and transforming growth factor-beta 3 cooperate to activate matrix metalloproteinase expression and invasiveness in A549 lung adenocarcinoma cells. *Cancer Letters*.

[28] Ardizzoni A., Baldini E., Mereu C. (1994). Biologic and clinical effects of continuous infusion interleukin-2 in patients with non-small cell lung cancer. *Cancer*.

[29] Tester W. J., Kim K. M., Krigel R. L. (1999). A randomized Phase II study of interleukin-2 with and without beta-interferon for patients with advanced non-small cell lung cancer. *Lung Cancer*.

[30] De Vita F., Turitto G., di Grazia M., Frattolillo A., Catalano G. (1998). Analysis of interleukin-2/interleukin-2 receptor system in advanced non-small-cell lung cancer. *Tumori Journal*.

[31] Chen Y.-M., Yang W.-K., Whang-Peng J. (1997). Restoration of the immunocompetence by IL-2 activation and TCR-CD3 engagement of the in vivo anergized tumor-specific CTL from lung cancer patients. *Journal of Immunotherapy*.

[32] Aznavoorian S., Murphy A. N., Stetler-Stevenson W. G., Liotta L. A. (1993). Molecular aspects of tumor cell invasion and metastasis. *Cancer*.

[33] Gho Y. S., Kim P. N., Li H. C., Elkin M., Kleinman H. K. (2001). Stimulation of tumor growth by human soluble intercellular adhesion molecule-1. *Cancer Research*.

[34] Qian Q., Zhan P., Yu L. (2011). Baseline levels and decrease in serum soluble intercellular adhesion molecule-1 during chemotherapy predict objective response and survival in patients who have advanced non-small-cell lung cancer. *Clinical Lung Cancer*.

[35] Ma J., Yu K. N., Cheng C., Ni G., Shen J., Han W. (2018). Targeting Nrf2-mediated heme oxygenase-1 enhances non-thermal plasma-induced cell death in non-small-cell lung cancer A549 cells. *Archives of Biochemistry and Biophysics*.

[36] Cao L., Suo X. J., Jiang W. (2019). [Effects of heme oxygenase-1 knockdown on proliferation, invasion and metastasis of lung adenocarcinoma A549 cells and its mechanism]. *Zhonghua Zhong Liu Za Zhi [Chinese Journal of Oncology]*.

[37] Perez-Gracia J. L., Prior C., Guillén-Grima F. (2009). Identification of TNF-*α* and MMP-9 as potential baseline predictive serum markers of sunitinib activity in patients with renal cell carcinoma using a human cytokine array. *British Journal of Cancer*.

[38] Shang G.-S., Liu L., Qin Y.-W. (2017). IL-6 and TNF-*α* promote metastasis of lung cancer by inducing epithelial-mesenchymal transition. *Oncology Letters*.

[39] Kessenbrock K., Plaks V., Werb Z. (2010). Matrix metalloproteinases: regulators of the tumor microenvironment. *Cell*.

[40] Wang H., Guan X., Tu Y. (2015). MicroRNA-29b attenuates non-small cell lung cancer metastasis by targeting matrix metalloproteinase 2 and PTEN. *Journal of Experimental & Clinical Cancer Research*.

[41] Rundhaug J. E., Mikulec C., Pavone A., Fischer S. M. (2007). A role for cyclooxygenase-2 in ultraviolet light-induced skin carcinogenesis. *Molecular Carcinogenesis*.

[42] Cao Y., Prescott S. M. (2002). Many actions of cyclooxygenase-2 in cellular dynamics and in cancer. *Journal of Cellular Physiology*.

